# Long-term work ability, cognitive impairments and work-related concerns among young adult cancer survivors: a comparative analysis with healthy controls (AYA-LE study)

**DOI:** 10.1186/s12885-026-15969-5

**Published:** 2026-04-07

**Authors:** Hannah Brock, Michael Friedrich, Kristina Geue, Anja Mehnert-Theuerkauf, Diana Richter, Annekathrin Sender, Katja Leuteritz

**Affiliations:** 1 Department of Medical Psychology and Medical Sociology, Comprehensive Cancer Center Central Germany (CCCG), University Medical Center Leipzig, Philipp-Rosenthal-Str. 55, Leipzig, 04103 Germany; 2 Medical Faculty, University Clinic of Psychosomatic Medicine and Psychotherapy, Otto- von-Guericke-University Magdeburg, Magdeburg, Germany

**Keywords:** Adolescent and young adult, Aya, Cancer, Psychooncology, Return to work, Work ability, Cognitive impairments, Work-related concerns

## Abstract

**Background:**

Cancer can substantially disrupt the career paths of adolescent and young adult cancer survivors (AYA-CS). However, little is known about the long-term impact of cancer on their work life compared to healthy peers without a history of cancer.

**Methods:**

As part of the AYA-LE study, we surveyed AYA-CS (aged 18–39 years at time of diagnosis) and healthy controls (HC). A multivariate analysis of covariance (MANCOVA) was conducted to compare work ability (Work Ability Index), cognitive impairments (COPSOQ) and work-related concerns between groups, controlling for differences in age, gender and educational level. Predictors of work ability were analyzed on average 4 years after diagnosis (t3) using hierarchical regression analyses.

**Results:**

A total of 438 AYA-CS and 406 HC participated in the study. AYA-CS reported significantly lower work ability (M = 35.73 ± 0.35 vs. M = 39.17 ± 0.35; *p* < 0.001, ηp² = 0.06) and more severe cognitive impairment (M = 34.88 ± 1.08 vs. M = 29.15 ± 1.10; *p* < 0.001, ηp² = 0.02) than HC. They also reported more work-related concerns about reduced income, unemployment, limited career advancement and early retirement (all *p* < 0.001). In AYA-CS, lower work ability at t3 was associated with older age, metastatic or recurrent disease, more severe cognitive impairment, greater effort to cope with the disease, less employer support, and participation in rehabilitation measures (adj. R² = 0.59), with cognitive impairment and effort to cope with the disease emerging as the strongest predictors of work ability.

**Conclusions:**

AYA-CS continue to experience poorer work outcomes than their peers without cancer, even years after diagnosis. Our findings emphasize the need for early identification of at-risk survivors and for interventions addressing cognitive impairments, coping demands, and workplace support to sustain and improve work ability in this population.

**Supplementary Information:**

The online version contains supplementary material available at 10.1186/s12885-026-15969-5.

## Introduction

In Germany, more than a third of cancer survivors are of working age [1]. Almost two-thirds of cancer patients either continue to work during treatment or return to work afterwards, making vocational reintegration a central issue even during treatment [2]. In particular, young adult cancer survivors who were diagnosed between the ages of 15 and 39 years – commonly referred to as Adolescents and Young Adults (AYA) by the National Cancer Institute [3] – have many years of working life ahead of them after completing treatment. Employment during this life stage is a key source of financial stability, social participation, and personal identity – factors that may be severely disrupted by a cancer diagnosis and its treatment [4].

Due to the intensive and prolonged nature of cancer therapy, AYA cancer survivors (AYA-CS) are often forced to interrupt their professional or academic activities during treatment, leading to absences, career disruptions, and financial strain [5–7]. Such work-related concerns are associated with increased psychological distress [8] and may persist or even intensify over time due to treatment-related physical or cognitive impairments, resulting in reduced working hours, occupational changes or even work disability [9, 10].

Because of their young age, employed AYA-CS may live longer with the late effects of cancer and its treatment and are therefore more likely to be affected by long-term and late consequences than survivors diagnosed in older stages of life [8]. Studies have shown that especially fatigue and cognitive impairments (CI) such as concentration difficulties, or memory problems can last for years after treatment and substantially affect occupational functioning [7, 11–13]. More than half of AYA-CS who had been employed full time before diagnosis reported cognitive problems 6–14 months and 15–35 months after diagnosis [8]. Patients experiencing CI appear to have the highest long-term risk of unsuccessful vocational and social (re-)integration, which in turn may adversely affect their quality of life [14].

Although most AYA-CS return to work after treatment, many report difficulties and delays in reintegration [6]. They also have higher rates of unemployment compared to healthy peers [7, 15–17]. However, employment rates provide only limited insight into actual work ability (WA), which is defined as the individual’s perceived capacity to meet professional demands [18]. To date, only few studies have focused on WA in AYA-CS [12, 19–21]. Previous work from our research group, conducted as part of a longitudinal study, demonstrated that more than 75% of AYA-CS reported reduced WA immediately after completing treatment, with 57% continuing to experience reduced WA one year later [20]. Similarly, Tan et al. found that 40% of AYA-CS reported that their WA was affected by lingering effects of cancer or its treatment two years after diagnosis [21]. Yet, data on the long-term course of WA beyond the first two years after diagnosis remain scarce [13]. Only one study examined AYA-CS 16 years post-diagnosis and found that 38% reported reduced WA [12]. Consequently, little is known about how WA develops in the long term – particularly in comparison to peers without a history of cancer. This is consistent with recent studies highlighting the need for longitudinal data and healthy control groups, and pointing out methodological limitations of previous research, where WA has frequently been assessed based on single item measures [12, 13, 20]. Comparison data are particularly crucial to distinguish cancer-related limitations in WA from age- or development-related differences.

In adult cancer survivors, persistent physical complaints, depression, fatigue, and CI have been associated with reduced WA [13, 22]. Among AYA-CS, only few studies have examined predictors of WA, but similar associations have been observed: a higher burden of treatment-related side effects and comorbidities, fatigue, depressive symptoms, as well as prolonged sick leave, lower basic education or parenthood were related to decreased WA [12, 20]. While work-related factors have been well examined among older cancer survivors [2, 13, 23], they have received little attention in AYA-CS studies to date [12, 20].

Therefore, the present study examined AYA-CS four years post-diagnosis to explore longer-term trajectories of WA. We aimed to systematically investigate the extent of WA, CI, and work-related concerns in AYA-CS compared to healthy controls (HC). Furthermore, we examined which sociodemographic, medical, and psychosocial (including work-related) factors are associated with long-term WA of AYA-CS.

Based on these aims, the following research questions were addressed:

1. Does the extent of work ability (WA) differ between AYA-CS and HC?
2. Does the extent of cognitive impairments (CI) differ between AYA-CS and HC?
3. Does the extent of work-related concerns differ between AYA-CS and HC?
4. Which sociodemographic, medical, and psychosocial factors are associated with work ability (WA) of AYA-CS four years after diagnosis?

## Methods

### Study design

The present study was conducted as part of the AYA-LE study (2014–2022), a nationwide prospective longitudinal study comprising six measurement points designed to examine life satisfaction, psychosocial care situation, and support needs among AYA-CS [24, 25]. The third assessment (t3), focusing specifically on the occupational situation of AYA-CS, was carried out between May and December 2018. At study entry, inclusion criteria for AYA-CS were: (1) a cancer diagnosis within the past four years (first manifestation, all malignant tumor entities, ICD-10: C00–C97), and (2) an age at diagnosis between 18 and 39 years. In addition, a comparison group of healthy young adults without a history of cancer (HC) was surveyed between January and February 2018. The study received approval from the Ethics Committee of the Faculty of Medicine, University of Leipzig (436/17-ek), and was funded by the German Cancer Aid.

### Recruitment

The recruitment of AYA-CS was carried out nationwide in cooperation with 16 acute oncology clinics, four rehabilitation clinics, and two tumor registries. Additionally, interested patients could self-register via social media (project website and Facebook). For the healthy comparison group, men and women from the general population aged 18 to 45 years – corresponding to the age range of the patient sample at the time of the survey – were recruited who had no history of cancer and were contacted via the local residents’ registration office. The recruitment strategy aimed to contact three individuals from the general population of the same age for each AYA-CS participant. This approach was intended to ensure that, assuming a conservative response rate of 40%, at least one comparison group participant would be available for each AYA-CS participant. After providing written informed consent to participate, both groups received either a link to a standardized online questionnaire (Lime Survey) or a printed version. To increase the participation rate, all study participants (AYA-CS and HC) received a compensation of €10 for each completed questionnaire. A detailed description of the recruitment procedure has already been published [24, 25].

### Instruments

#### Work Ability Index short version (WAI-r)

The German short version of the Work Ability Index (WAI-r) was used to assess work ability in AYA-CS and HC [18, 26]. The WAI is a widely used and validated instrument that integrates the worker’s health status, personal resources, and the physical as well as psychological demands of their job. It captures the extent to which an employee is able to perform work tasks in view of their individual circumstances and working conditions [27]. Validation studies from large Finnish longitudinal cohorts have demonstrated its high predictive validity for future work ability and satisfactory internal consistency (Cronbach’s α = 0.83) [27, 28].

The WAI comprises seven items assessing: (1) current work ability compared with lifetime best, (2) job demands, (3) current diseases, (4) perceived work impairment, (5) sick leave, (6) prognosis of work ability in two years, and (7) mental resources. The short version differs from the original version only with respect to Item 3. Whereas original version includes a checklist of 51 specific diseases, the short version assesses 14 aggregated disease groups. Using a slightly modified scoring procedure, the short version yields total scores that are almost identical to those obtained with the long version [26, 29]. The overall WAI score is calculated using a standardized algorithm integrating all seven domains and ranges from 7 to 49 points. According to Ilmarinen (2009), total scores can be classified into four categories: poor (7–27), moderate (28–36), good (37–43), and excellent (44–49) [18]. Work ability in relation to physical and mental demands of work was rated on a 5-point Likert scale ranging from 1 = very poor to 5 = very good.

#### Copenhagen Psychosocial Questionnaire (COPSOQ)

The subjective extent of cognitive impairments was assessed using the four-item cognitive impairments subscale of the Copenhagen Psychosocial Questionnaire (COPSOQ), a validated tool for measuring mental stress at work [30]. This subscale was preferred over the full COPSOQ for economic reasons. The German version demonstrates good internal consistency (Cronbach’s α = 0.85 – 0.87) [31]. Participants rated the following items on a 5-point Likert scale (1 = always to 5 = never/hardly ever): “How often during the past four weeks have you (1) had problems with concentration, (2) had difficulty with making decisions, (3) had difficulty with remembering, and (4) found it difficult to think clearly?”. As recommended, the mean score across the four items was calculated after reverse-coding the response scale and subsequently standardized to a 0–100 scale (0–20 = never/hardly ever, 21–40 = seldom, 41–60 = sometimes, 61–80 = often, 81–100 = always), with higher values indicating greater cognitive load, allowing comparison with previous studies.

#### Perceived Adjustment to Chronic Illness Scale (PACIS)

The effort coping with the disease was evaluated using the single-item Perceived Adjustment to Chronic Illness Scale (PACIS) [32]. Participants were asked to rate the question, “How much effort does it cost you to cope with your illness?” on a scale ranging from 0 (“none”) to 100 (“a great deal”). This measure was therefore administered exclusively to AYA-CS and not to HC without a history of cancer. The PACIS item serves as a global measure of disease management, is appropriate for use in clinical trials, and has been validated across diverse cancer populations [32].

#### Work-related concerns and employer support

We used a German multidimensional questionnaire developed and psychometrically validated by Bürger et al., which assesses the conditions for reintegration into work following a disease. It takes into account both the personal relevance of working activities and the employers perspective (and others, e.g. family, physicians) on the patients employment [33, 34]. Originally developed and validated in orthopedic patients, it can also be applied to other disease groups, such as individuals with cancer [35]. Work-related concerns were assessed with four items addressing potential future work-related consequences. Patients were asked: “How often do you worry about (1) earning less money, (2) becoming unemployed, (3) having fewer opportunities for advancement and career, and (4) retiring early in the future because of your health condition?”. Responses were rated on a 4-point Likert scale ranging from 0 (“almost never”) to 3 (“very often”).

Another questionnaire item was used to assess support from the employer. Participants were asked to rate the statement, “I receive all necessary support from my employer to return to work as quickly as possible,” on a 5‑point Likert scale ranging from 0 (“not at all”) to 4 (“completely”).

#### Sociodemographic and medical data

General sociodemographic and medical data were collected at t3 using self-developed items. AYA-CS could answer “yes” or ‘no’ to the question “Have you received any follow-up treatment or oncological rehabilitation since your cancer diagnosis?”.

### Statistical analysis

Statistical analyses were performed using IBM SPSS Statistics 29. Descriptive statistics were computed to characterize the sample. Missing data were handled using the expectation - maximization algorithm where applicable. Cases for which imputation was not feasible were excluded from the respective analyses (complete-case analyses) [36]. Group differences between AYA-CS and healthy controls (HC) were tested using chi-square tests, with effect sizes quantified using the phi coefficient (φ) and interpreted as small (0.1), medium (0.3), and large (0.5) [37]. Differences between physical and mental work ability among AYA-CS were analyzed using a paired t-test.

To control for sociodemographic differences between groups, a multivariate analysis of covariance (MANCOVA) was conducted with group (AYA-CS vs. HC) as the fixed factor and age, gender, and educational level entered as covariates. The dependent variables included WA, CI and work-related concerns (four items), which were analyzed jointly within the multivariate model. Homogeneity of variances was assessed using Levene’s test, and homogeneity of variance-covariance matrices was evaluated using Box’s M test. Follow-up univariate analyses were conducted to aid interpretation of multivariate results. Effect sizes were calculated as partial eta squared (ηp²) and interpreted according to Cohen’s conventions: small (0.01), medium (0.06), and large (0.14) [37].

Blockwise hierarchical regression analyses were performed to identify predictors of WA among AYA-CS at t3 (see below). Independent variables at t3 were selected based on significant correlations with the dependent variable (*r* > 0.10) and prior literature on older cancer patients [13, 23, 38]. Employer support was included due to the limited exploration of work-related factors in previous research among AYA-CS. The correlation matrix of all measured variables and their associations with WA in AYA-CS is provided in the Supplementary Material (Supplementary Table S1). Multicollinearity was checked prior to the analyses, with all VIFs between 1.04 and 1.33. Variables were entered in three blocks:

- Block I: Sociodeographic variables: sex (male/female), age, monthly net household income (< €3000/≥ €3000)
- Block II: Medical variables: metastatic or recurrent disease (no/yes), stem cell transplantation (no/yes).
- Block III: Psychosocial variables: COPSOQ (0–100), PACIS (0–100), employer support (not at all/partly vs. mostly/completely), follow-up treatment/rehabilitation (no/yes).

## Results

### Sample characteristics

After screening for eligibility, *N* = 762 AYA-CS patients provided written informed consent. Following *N* = 185 dropouts, the sample size at the initial assessment (t1) was *N* = 577. Between t1 and t3, the dropout rate was 24% (*N* = 139), resulting in a final sample of *N* = 438 AYA-CS patients at t3. Dropouts were slightly younger at diagnosis (M = 28.4, SD = 5.8) than completers (M = 29.6, SD = 6.2; t = − 2.094, *p* = 0.037) and were more often male (33.1% vs. 24.4%, χ²(1, 577) = 4.01, *p* = 0.044). No differences were observed with respect to time since diagnosis, parenthood, educational level, or work ability (WAI Item 1). Of the *N* = 1598 healthy controls (HC) contacted, *N* = 421 responded, and *N* = 406 (25.5%) provided written informed consent.

Sociodemographic, medical, and psychosocial characteristics of the final sample are summarized in Table 1. Nearly 75% of the AYA-CS participants were female, with a mean age of M = 33.8 years. The most common cancer types were breast cancer (25.8%), Hodgkin’s lymphoma (18.9%), and gynecological cancer (9.4%). The mean time since diagnosis was approximately four years (M = 50.1 months, SD = 9.7). Participants in the healthy control group were significantly younger (*p* < 0.001), more frequently male (*p* < 0.001) and reported a higher educational level (*p* = 0.003) compared to the AYA-CS group.

**Table 1:** Sociodemographic, medical and psychosocial characteristics AYA-CS and HC (t3)

	AYA-CS (*N* = 438)	HC (*N* = 406)	
	*N* (%)	*N* (%)	*p* ^d^
*Sociodemographic characteristics*			
Age at diagnosis (M, SD)	29.6 (6.2)	-	-
Age (M, SD)	33.8 (6.3)	31.4 (5.8)	**< 0.001**
Gender			
Male	110 (25.1)	154 (37.9)	**< 0.001**
Female	328 (74.9)	252 (62.1)	
Highest educational degree			
No school completion/≤10 years	163 (37.2)	114 (28.1)	**0.003**
> 10 years	265 (60.5)	286 (70.4)	
Relationship			
Yes	327 (74.6)	305 (75.1)	0.914
No	108 (24.7)	99 (24.4)	
Children (yes)			
Yes	181 (41.3)	163 (40.2)	0.796
No	257 (58.7)	240 (59.6)	
Employed or studying	366 (83.6)	357 (87.9)	0.057
Not employed	72 (16.4)	48 (11.8)	
Monthly net income per household			
< €3000	220 (50.2)	210 (51.7)	0.740
≥ €3000	177 (40.4)	161 (39.7)	
Recruitment			
Rehabilitation clinics	165 (37.7)	-	-
Acute care hospitals	48 (11.0)	-	-
Tumor registries	133 (30.4)	-	-
Self-registration (e.g., homepage, e-mail)	92 (21.0)	-	-
*Medical characteristics*			
Time since diagnosis in months (M, SD)	50.1 (9.7)	-	-
Cancer type			
Breast cancer	113 (25.8)	-	-
Gynecological cancer	41 (9.4)	-	-
Testicular cancer	36 (8.2)	-	-
Thyroid cancer	28 (6.4)	-	-
Hodgkin’s lymphoma	83 (18.9)	-	-
Non-Hodgkin’s lymphoma	26 (5.9)	-	-
Hematological cancer	30 (6.8)	-	-
Other	81 (18.5)	-	-
Solid tumor	299 (68.3)	-	-
Therapy^a^			
Chemotherapy^b^	268 (61.2)	-	-
Radiotherapy^c^	168 (38.4)	-	-
Surgery	278 (63.5)	-	-
Stem cell transplant	23 (5.3)	-	-
*Psychosocial characteristics*			
Effort coping with the disease (PACIS; M, SD)	24.8 (28.0)	-	-

Percentages may not add up to 100% due to missing data

Independent samples t-tests and chi square tests were carried out to compare AYA-CS with HC

Bold type indicates statistical significance

*M* Mean, *SD* standard deviation, *PACIS* Perceived Adjustment to Chronic Illness Scale (scale 0-100)

^a^ multiple answers possible

^b^ includes radiochemotherapy

^c^ includes radiochemotherapy and nuclear therapy

^d^ type-I-error probability

### Work ability (WA)

Levene’s test indicated significant inequality of variances across the dependent variables, and Box’s M test was also significant (Box’s M = 105.17, F = 4.97, df1 = 21, df2 = 2,132,367.824, *p* < 0.001). Accordingly, Pillai’s trace was used as the primary multivariate test. The MANCOVA, controlling for age, gender, and educational level, revealed a significant multivariate effect of group on the combined dependent variables (WA, CI, and work-related concerns), Pillai’s trace V = 0.078, F(6, 755) = 10.66, *p* < 0.001, ηp² = 0.08.

**Fig. 1:**
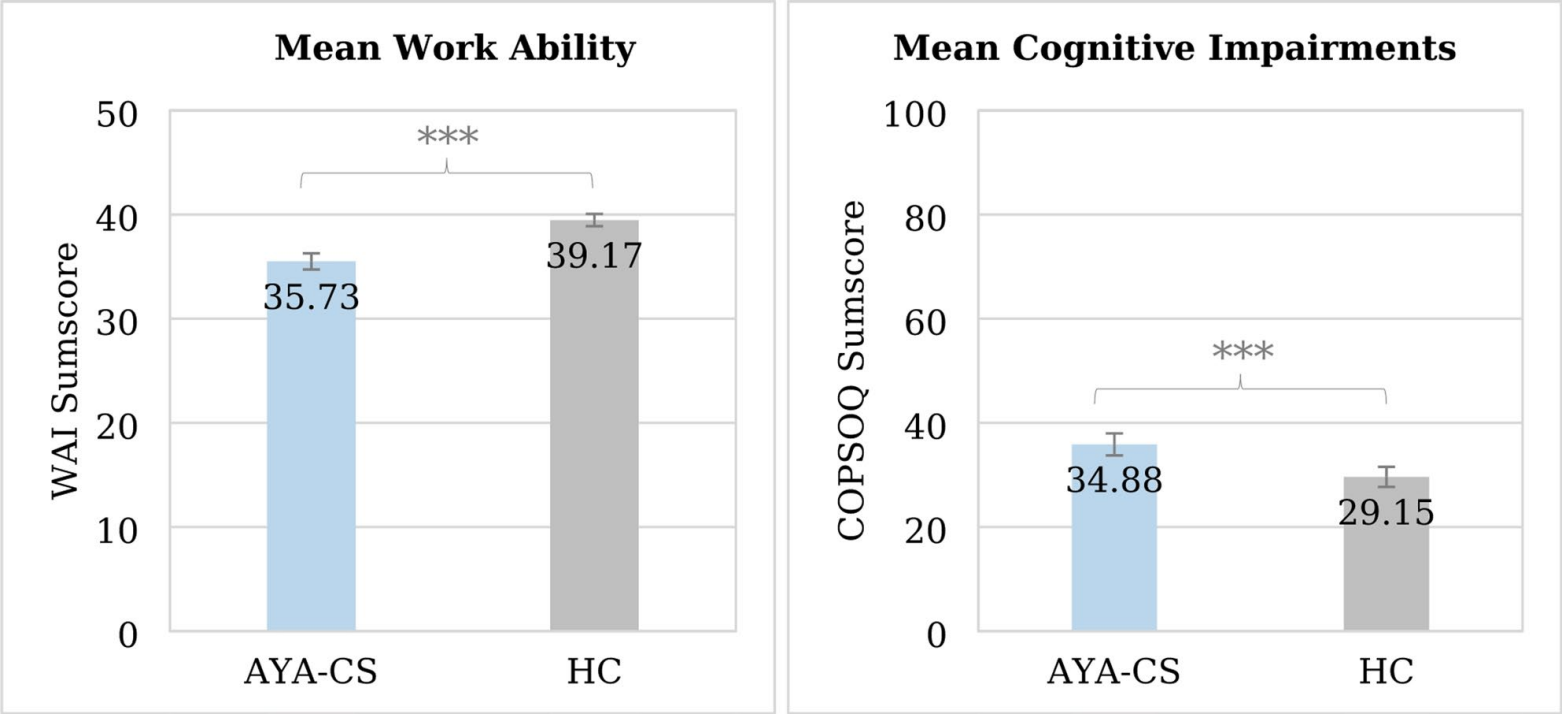
Adjusted mean work ability and cognitive impairments of AYA-CS and HC measured with the Work Ability Index (WAI) and the Copenhagen Psychosocial Questionnaire (COPSOQ). Error bars represent 95% confidence intervals. *** *p* < 0.001

Follow-up univariate tests indicated a significant difference in WA between AYA-CS and HC at t3, F(1, 760) = 47.20, *p* < 0.001 (see Table 2; Fig. 1). This difference can be described as a medium effect (ηp² = 0.06). Furthermore, 48.3% (*n* = 189) of AYA-CS reported having moderate to critical, and thus reduced, work ability, compared to 27.2% (*n* = 101) of HC (χ²(1) = 36.00, *p* < 0.001, φ = 0.21).

The majority of AYA-CS (61.0%, *n* = 267) perceive their work as more mentally than physically demanding, while 8.2% (*n* = 36) perceive it as more physically than mentally demanding, and 22.1% (*n* = 97) rate both demands as equally strong. The current work ability of AYA-CS with regard to physical demands (M = 3.9, SD = 1.0) does not differ significantly from their current work ability with regard to mental demands (M = 3.8, SD = 1.0), t(411) = 0.64, *p* = 0.521.

### Cognitive impairments (CI)

The adjusted mean CI score at t3 was significantly higher in AYA-CS (M = 34.88, SE = 1.08) than in HC (M = 29.15, SE = 1.10), F(1, 760) = 13.41, *p* < 0.001 (see Table 2). This difference represents a small effect (ηp² = 0.02; see Fig. 1). Of AYA-CS, 15.5% (*n* = 68) reported a CI score > 60 compared to 6.9% (*n* = 28) in HC (χ²(1) = 15.56, *p* < 0.001, φ = 0.14).

### Work-related concerns

Compared to HC, AYA-CS reported significantly more concerns about potential negative career and income consequences due to their health condition (see Table 2). Specifically, AYA-CS reported greater worry about reduced income, unemployment, limited career advancement and early retirement. The magnitude of effect sizes can be classified as small (all ηp² ≤ 0.05).

**Table 2:** Multivariate test statistics and adjusted means from the MANCOVA

		AYA-CS^a^		HC^a^					
		M^b^	SE	M^b^	SE	F	df1, df2	*p*	ηp²
Work Ability (WAI)		35.73	0.35	39.17	0.35	47.20	1, 760	**< 0.001**	0.06
CI (COPSOQ)		34.88	1.08	29.15	1.10	13.41	1, 760	**< 0.001**	0.02
Work-related concerns									
Reduced income		1.07	0.05	0.64	0.05	38.50	1, 760	**< 0.001**	0.05
Unemployment		0.88	0.05	0.63	0.05	13.96	1, 760	**< 0.001**	0.02
Limited career advancement		1.00	0.05	0.70	0.05	18.05	1, 760	**< 0.001**	0.02
Early retirement		0.75	0.04	0.43	0.04	28.44	1, 760	**< 0.001**	0.04

Bold type indicates statistical significance

*WAI* Work Ability Index (scale 7–49), *CI* Cognitive Impairments, *COPSOQ* Copenhagen Psychosocial Questionnaire (scale 0–100), *M* Mean, *SE* Standard error, *F* F-statistic (MANCOVA), *df1* numerator degrees of freedom, *df2* denominator degrees of freedom, *p* type-I-error probability, *ηp²* partial eta-squared

^a^ MANCOVA sample comprised *n* = 391 AYA-CS and *n* = 374 HC due to missing data

^b^ Means represent estimated marginal means (EMMs), adjusted for all covariates included in the MANCOVA

### Associations with work ability at t3

Results from hierarchical linear regression models examining the independent associations of sociodemographic, medical, and psychosocial factors with WA of AYA-CS at t3 are summarized in Table 3. Block I (sociodemographic characteristics) explained a small but significant portion of variance (adjusted R² = 0.087). The addition of medical characteristics in Block II further increased the explained variance (ΔR² = 0.140). Block III, comprising psychosocial characteristics, added substantial explanatory power (ΔR² = 0.370).

**Table 3:** Hierarchical linear regression analysis for work ability among AYA-CS at t3

F-Statistics	Model 1			Model 2			Model 3		
	F(3, 256) = 9.275, *p* < 0.001			F(5, 254) = 15.897, *p* < 0.001			F(9, 250) = 43.233, *p* < 0.001		
adj. R^2^	0.087			0.223			0.595		
ΔR^2^				0.140			0.370		
	B	β	p-value	B	β	p-value	B	β	p-value
Block I - Sociodemographic characteristics									
Sex^1^	–2.465	–0.148	0.015	–2.131	–0.128	0.023	–0.413	–0.025	0.547
Age	–0.212	–**0.176**	**0.004**	–0.220	–**0.182**	**0.001**	–0.141	–**0.116**	**0.005**
Monthly net income per household^2^	3.157	0.215	< 0.001	3.296	0.224	< 0.001	1.060	0.072	0.086
Block II - Medical characteristics									
Metastatic or recurrent disease^3^				–8.796	–**0.353**	**< 0.001**	–5.082	–**0.204**	**< 0.001**
Stem cell transplant^4^				–2.340	–0.070	0.231	–1.791	–0.053	0.210
Block III - Psychosocial characteristics									
Cognitive impairments (COPSOQ)^5^							–0.103	–**0.323**	**< 0.001**
Effort coping with the disease (PACIS)^6^							–0.087	–**0.324**	**< 0.001**
Employer support^7^							3.322	**0.219**	**< 0.001**
Follow-up medical treatment/rehabilitation^8^							–2.085	–**0.124**	**0.002**

Bold type indicates statistical significance

*F* F-statistic, *p* type-I-error probability, *adjusted R*_2_ variance explained by the model, Δ*R*_2_change in R_2_, *B *unstandardized regression coefficient, β standardized beta coefficient, *p *type-I-error probability, *CI *95% confidence interval, reference categories

_1_ male, _2_ < €3000, _3_ no, _4_ no, _5, 6_ 0–100, _7_ “I receive all the necessary support from my employer to return to work quickly – *not at all/partly*.”, _8_ no

Due to listwise deletion of cases with missing data on predictors, the regression analysis was conducted on *n* = 260 AYA-CS

In the final model (Model 3), lower WA at t3 was significantly associated with sociodemographic factors (higher age), medical factors (presence of a metastatic or recurrent disease), and psychosocial factors (greater CI, greater effort coping with the disease, lower employer support, and participation in follow-up medical treatment or rehabilitation). Among psychosocial variables, COPSOQ and PACIS exhibited the largest standardized regression coefficients (β = −0.32, *p* < 0.001). A 10-point increase in the COPSOQ was associated with an approximately 1-point decrease in WA (B = − 0.103), while a 10-point increase in the PACIS corresponded to a 0.9-point decrease in WA (B = − 0.087). The final model explained 59% of the variance in WA at t3 (adjusted R² = 0.59) and demonstrated significant predictive ability, F(9, 250) = 43.23, *p* < 0.001 (see Table 3).

## Discussion

This nationwide case–control study examined and compared WA, CI, and work-related concerns between AYA-CS and HC. Furthermore, the study aimed to identify long-term risk factors for reduced WA at t3. To our knowledge, this is the first study to compare WA between AYA-CS and HC using validated tools.

### Work ability

AYA-CS reported significantly lower WA than HC. Nearly half of AYA-CS indicated reduced WA compared to less than one-third of controls. This suggests that even years after diagnosis, many AYA-CS do not regain age-appropriate WA, highlighting that employment status alone does not reflect actual capacity to meet occupational demands.

In line with previous results from the same cohort [20], the proportion of AYA-CS with reduced WA decreased over time – from 76% immediately after treatment to 57% two years and 48% four years post-diagnosis – yet remains considerably higher than in healthy peers. It should be noted that earlier assessments relied on a single WAI item, limiting comparability. Similarly, Tan et al. found that 40% of AYA-CS reported impaired WA two years after diagnosis, though their study was limited by small sample size, lack of controls, and non-validated measures [21].

Evidence from older cancer populations shows comparable patterns of reduced WA among cancer survivors compared to HC [13, 39, 40]. Consistent with our findings, recent research also reports lower employment rates, working hours, income losses, and increased disability benefits among AYA-CS compared to HC, which may further reflect diminished WA [15–17, 41]. These impairments may be related to the intensive, systemic treatments typical for AYA-CS due to their young age and tumor biology, which heighten risks for long-term physical and psychosocial sequelae [11, 12, 15]. Consequently, survivorship care should not only facilitate return to work but ensure sustainable vocational reintegration through ongoing support to maintain and enhance WA.

### Cognitive impairments

The extent of CI was significantly higher among AYA-CS compared to HC. Evidence on CI in AYA-CS remains limited, as most studies have focused on other age groups. However, our findings align with those of Dewar et al., who also reported higher rates of cognitive dysfunction among AYA-CS relative to the general population [42]. Psychological comorbidities such as anxiety and depression, which frequently persist in this population, further exacerbate subjectively perceived cognitive difficulties [10]. In addition to subjective complaints, AYA-CS also perform worse on objective neurocognitive tests than healthy peers [14]. Given that brain development is often still ongoing during adolescence and young adulthood, AYA-CS are considered particularly vulnerable to cognitive dysfunction [43], which may explain the long-term adverse effects of cancer treatment on neurological development [44]. A recent scoping review estimated the mean prevalence of CI in AYA-CS to be around 26%, with symptoms persisting for up to 15 years post-diagnosis [10]. Such persistent cognitive problems can substantially affect daily functioning, e.g. by impairing educational attainment or employment opportunities [10], and may in turn negatively impact psychological well-being [11]. Overall, these findings indicate that AYA-CS are at increased risk of CI compared to HC, even years after treatment completion, underscoring the need for targeted support addressing CI in this population. Evidence suggests that cognitive training, as well as multimodal interventions combining cognitive, physical, and psychological approaches, may improve CI, although larger, more robust trials are needed [45, 46].

### Work-related concerns

In summary, AYA-CS perceive their occupational situation as more precarious than HC. Even on average four years after diagnosis, they report more frequent concerns about finances, employment status, career prospects, and early retirement. The overall burden of work-related concerns is up to twice as high as among HC.

International comparisons are limited due to the scarcity of studies including control groups and methodological differences, as worries were often assessed with self-developed items, and the questionnaire used in our study has only been applied in German-speaking contexts. Nonetheless, our findings align with previous research indicating that AYA-CS experience greater financial difficulties and are more likely to receive disability benefits than HC [6, 15]. Other quantitative and qualitative studies have shown that financial and occupational concerns are among the strongest determinants of reduced life satisfaction in AYA-CS [47, 48]. In a Canadian study, such concerns were even more pronounced in AYA-CS than in older cancer survivors, likely reflecting age-related factors such as temporary employment and limited financial reserves [49]. Work-related concerns have been shown to negatively affect both mental and physical health and to impair quality of life in AYA-CS [48, 50, 51], highlighting the enduring impact of cancer and its treatment on the occupational and psychosocial wellbeing of this population.

#### Associations with long-term work ability

Among the examined variables, age, metastatic or recurrent disease, CI, effort coping with the disease, and participation in rehabilitation showed significant negative associations with WA at t3, whereas employer support was positively associated. Psychosocial factors – particularly CI and effort coping with the disease – emerged as the strongest predictors of long-term reduced WA among AYA-CS.

Older AYA-CS reported lower WA compared with younger ones, which may reflect higher occupational and family responsibilities as well as cumulative disease and treatment-related late effects. We cannot completely rule out that the observed age effect is partly influenced by selection bias, as completers and dropouts differed slightly in age at diagnosis.

The negative association between a metastatic or recurrent disease and WA can be explained by the higher treatment intensity and disease burden typically associated with advanced cancer. Repeated or combined systemic therapies and the need to confront one’s own mortality often led to persistent physical and psychological side effects that may impair WA. Previous studies have reported similar findings: greater treatment intensity was linked to lower WA and a higher risk of unemployment [12, 15]. Likewise, studies in older cancer populations indicate that the presence of metastases is associated with a lower likelihood of returning to work [23].

Comparable findings have been reported for cancer-related fatigue, which frequently persists among AYA-CS and correlates negatively with WA [12, 39]. Quantitative and qualitative evidence further confirms that CI significantly affect work performance [8, 48]. Attention and memory problems may account for up to 40% of the variance in WA [13]. These results underscore the enduring support needs of AYA-CS facing CI and related long-term symptoms.

The finding that AYA-CS who reported greater efforts to cope with their disease had lower WA may suggest that intense coping reflects elevated psychological distress. Anxiety and depression are among the most common causes of reduced WA [12, 52]. AYA-CS must cope with cancer during a life phase characterized by career establishment and personal life planning, which may exacerbate psychological strain. Previous research shows that AYA-CS experience higher levels of distress compared with older cancer patients [53]. Promoting adaptive coping strategies and psychological stability therefore appears essential to maintain long-term WA in this group.

The negative association between rehabilitation participation and WA is likely attributable to selection effects. AYA-CS who opt for rehabilitation may already experience greater physical and psychological burden and, consequently, lower WA prior to the intervention.

Employer support emerged as a protective factor. Prior studies in older cancer populations have shown that support from supervisors and colleagues facilitates the return to work process [23, 54], whereas insufficient support is associated with lower WA [39]. A positive organizational climate and supportive leadership style are particularly beneficial for women [13, 23], who also represent the majority of our sample. Employers who communicate openly and actively support their employees may therefore play a crucial role in sustaining the long-term WA of AYA-CS.

### Limitations

Although this study was conducted in a large sample, several limitations should be considered when interpreting the results. First, female gender and higher educational levels were overrepresented in both AYA-CS and HC, reflecting a common phenomenon, as men are generally less likely to participate in clinical trials [55]. Second, CI and work-related concerns were assessed using instruments that have not yet been specifically validated in cancer populations [30, 33, 34]. While these measures capture relevant aspects of work reintegration and cognitive functioning, their psychometric properties in oncological contexts remain to be confirmed. Third, missing data led to reduced sample sizes for the MANCOVA and the regression analyses. Although the remaining sample sizes were sufficient for the conducted analyses, statistical power and generalizability may have been limited. Fourth, although mean differences in WA and CI between AYA-CS and HC were statistically significant, the observed effect sizes were small to medium. Accordingly, these findings should be interpreted with caution, as statistical significance in large samples does not imply clinical relevance at the individual level. Finally, employer support was measured using a single-item indicator. While this approach provides a pragmatic estimate of perceived support, more comprehensive and psychometrically robust instruments could offer a better understanding of workplace factors influencing WA.

### Implications for research and practice

The findings of this study indicate that AYA-CS may be at risk for long-term impairments in WA and CI, suggesting that early identification could inform targeted support. Future research should examine the underlying causes of these limitations and evaluate effective strategies to improve employment outcomes in at-risk groups [16], with particular attention to work-related factors. In this context, exploring contextual and subgroup-specific moderators (e.g., disease characteristics, occupational context, or institutional factors) may further help to tailor interventions to the specific needs of AYA-CS.

Although various multimodal interventions have shown potential to support work participation and related outcomes in adult cancer survivors, further research is needed to determine which measures can sustainably enhance WA, CI, and coping skills in AYA-CS [56]. To date, only a few evaluated approaches specifically target this age group. Hauken et al. demonstrated that a multidimensional, goal-oriented rehabilitation program tailored to AYA-CS – combining peer support, individualized multidisciplinary follow-up, psychoeducation, and physical activity – can enhance goal performance and satisfaction, coping, and return-to-work outcomes among participants [57]. Digital interventions may also represent a practical and effective option for AYA-CS but require further systematic evaluation [10].

As not all AYA-CS fully recover in cognitive functioning, supporting work participation should extend beyond individually targeted interventions. Concepts such as job crafting, combined with organizational accommodations, may help align job demands with individual resources and share responsibility between employees and employers [58, 59]. In practice, employment-related issues could be addressed through flexible working arrangements, vocational counseling, targeted training programs [60], and employer education to recognize psychosocial needs, offer gradual return-to-work plans, task adjustments, or ergonomic adaptations [23].

The results highlight the need of interprofessional collaboration among healthcare providers, patients, and employers to identify barriers early and develop individualized solutions [54]. Social work support and age-specific, vocationally oriented rehabilitation programs should be expanded and made available in the long term, especially since future career prospects are considered very important by AYA-CS [61]. Survivorship programs provide an appropriate framework to identify and support at-risk patients over time, as many AYA-CS perceive vocational challenges only later in survivorship or feel overwhelmed by multiple specialized services [11, 54].

At the same time, systemic and policy-level changes are required to ensure sustainable funding for such services. From a societal perspective, facilitating the vocational reintegration of AYA-CS is essential, given that the indirect costs of cancer in Germany (≈ €18.5 billion per year) – including work disability and reduced productivity – are comparable to direct medical costs [62]. This need becomes even more pressing in the context of an ageing society, where maintaining workforce participation and productivity is of growing importance.

## Conclusions

A successful return to work represents a central and existential topic for AYA-CS. Our findings indicate that while the majority of AYA-CS are employed in the long term, they report lower WA and more pronounced CI compared to healthy peers. These difficulties may reflect financial pressure or concerns about job security, both of which were also more pronounced among AYA-CS compared to HC. Early identification of at-risk survivors, the targeted development of interventions to strengthen cognitive and psychological resources, and the provision of flexible, workplace-based support are therefore essential. Interprofessional collaboration among oncology professionals, social workers, rehabilitation services, and employers may play a key role in facilitating return to work and sustaining WA over time.

## Supplementary Information

Supplementary Material 1.

## Data Availability

The datasets generated and analyzed during the current study are available from the corresponding author on reasonable request.

## Abbreviations

AYA-CS: Adolescent and young adult cancer survivors
HC: Healthy controls
WA: Work ability
CI: Cognitive impairments
