# Supplementary material for: Long-term work ability, cognitive impairments and work-related concerns among young adult cancer survivors: a comparative analysis with healthy controls (AYA-LE study)
Source: BMC Cancer. 2026 Apr 7;26:481. doi: 10.1186/s12885-026-15969-5 (PMC13081537; doi:10.1186/s12885-026-15969-5)
Supplement: Supplementary file 1 — Supplementary Material 1. [file 12885_2026_15969_MOESM1_ESM.docx]

**Supplementary Table S1.** Correlation matrix of all measured variables and work ability in AYA-CS

|  |  | 1 | 2 | 3 | 4 | 5 | 6 | 7 | 8 | 9 | 10 | 11 | 12 | 13 | 14 | 15 | 16 | 17 |
| --- | --- | --- | --- | --- | --- | --- | --- | --- | --- | --- | --- | --- | --- | --- | --- | --- | --- | --- |
| 1 | Work Ability | - |  |  |  |  |  |  |  |  |  |  |  |  |  |  |  |  |
| 2 | Sex^b^ | **-0.173^a^** | - |  |  |  |  |  |  |  |  |  |  |  |  |  |  |  |
| 3 | Age | **-0.174^a^** | **0.137** | - |  |  |  |  |  |  |  |  |  |  |  |  |  |  |
| 4 | Monthly net income per household^c^ | **0.186^a^** | 0.030 | 0.092 | - |  |  |  |  |  |  |  |  |  |  |  |  |  |
| 5 | Education level^d^ | -0.032 | 0.054 | -0.010 | 0.095 | - |  |  |  |  |  |  |  |  |  |  |  |  |
| 6 | Partnership^e^ | 0.064 | 0.074 | **0.178** | **0.405** | -0.013 | - |  |  |  |  |  |  |  |  |  |  |  |
| 7 | Children^f^ | 0.022 | 0.101 | **0.502** | **0.291** | -0.084 | **0.335** | - |  |  |  |  |  |  |  |  |  |  |
| 8 | Time since diagnosis | -0.093 | -0.051 | **0.269** | -0.044 | **0.159** | -0.072 | 0.094 | - |  |  |  |  |  |  |  |  |  |
| 9 | Surgery^g^ | -0.080 | **0.131** | 0.073 | -0.015 | 0.097 | 0.094 | 0.026 | -0.046 | - |  |  |  |  |  |  |  |  |
| 10 | Chemotherapy^h^ | -0.033 | **-0.175** | **-0.136** | -0.016 | 0.048 | -0.023 | -0.066 | 0.091 | **0.211** | - |  |  |  |  |  |  |  |
| 11 | Radiation^i^ | -0.088 | **0.129** | **0.151** | 0.065 | **0.128** | **0.107** | **0.132** | **0.124** | **0.229** | **0.346** | - |  |  |  |  |  |  |
| 12 | Stem cell transplant^j^ | **-0.147^a^** | -0.081 | -0.087 | **-0.170** | -0.010 | -0.095 | -0.045 | 0.053 | **-0.125** | **0.176** | 0.010 | - |  |  |  |  |  |
| 13 | Metastatic/recurrent disease^k^ | **-0.361^a^** | 0.098 | 0.012 | 0.076 | **0.123** | -0.075 | 0.070 | 0.017 | 0.077 | **0.145** | 0.069 | **0.182** | - |  |  |  |  |
| 14 | Cognitive Impairments (COPSOQ)^l^ | **-0.565^a^** | **0.164** | 0.017 | **-0.189** | -0.002 | -0.067 | 0.001 | 0.042 | 0.053 | 0.010 | 0.063 | 0.071 | **0.117** | - |  |  |  |
| 15 | Effort coping with the disease (PACIS)^m^ | **-0.581^a^** | **0.162** | **0.121** | **-0.118** | -0.055 | 0.044 | 0.029 | -0.015 | 0.058 | 0.016 | 0.063 | 0.007 | **0.275** | **0.399** | - |  |  |
| 16 | Employer support^n^ | **0.390^a^** | -0.032 | **-0.107** | **0.107** | -0.014 | 0.010 | -0.022 | 0.025 | 0.004 | -0.035 | -0.068 | **-0.172** | -0.083 | **-0.221** | **-0.128** | - |  |
| 17 | Follow-up medical treatment/ rehabilitation^o^ | **-0.263^a^** | 0.022 | -0.037 | **-0.106** | 0.049 | **-0.120** | **-0.191** | -0.002 | **0.129** | **0.205** | 0.061 | 0.023 | 0.094 | **0.126** | **0.152** | -0.097 | - |

Pearson correlation coefficients (r) are shown. Bold type indicates statistical significance.

^a^Variables that met the a priori inclusion criterion (r ≥ 0.10 with work ability) are shown in bold type and were included in the regression analyses.

reference categories: ^b^ male, ^c^ < €3000, ^d^ no school completion/≤10 years, ^e, f, g, h, I, j, k, o^ no, ^l, m^ 0 – 100, ^n^ “I receive all the necessary support from my employer to return to work quickly – *not at all/partly*.”.
